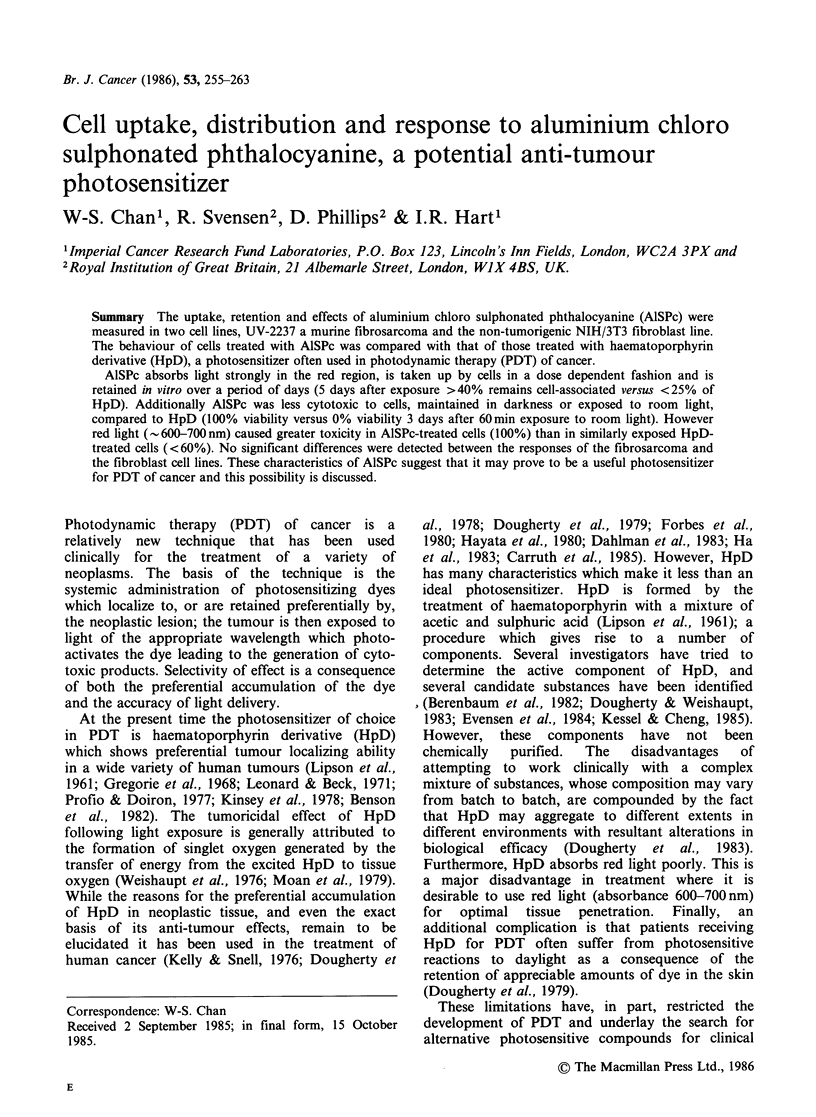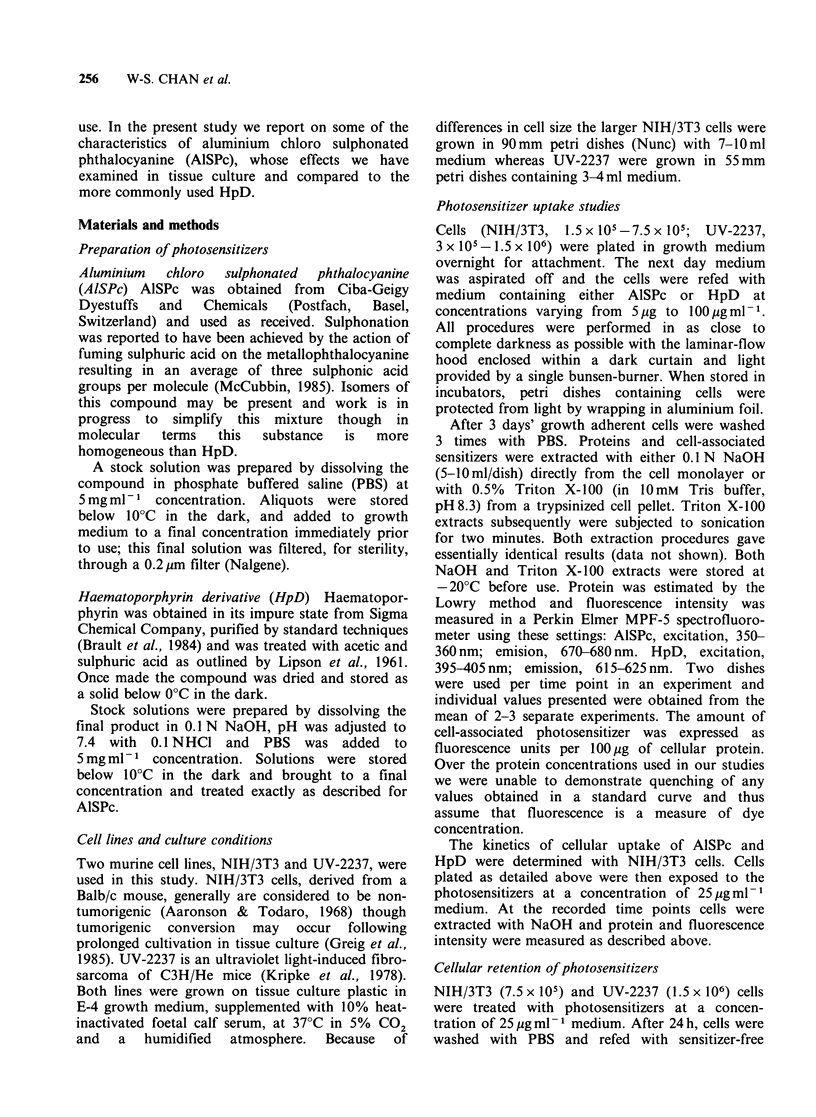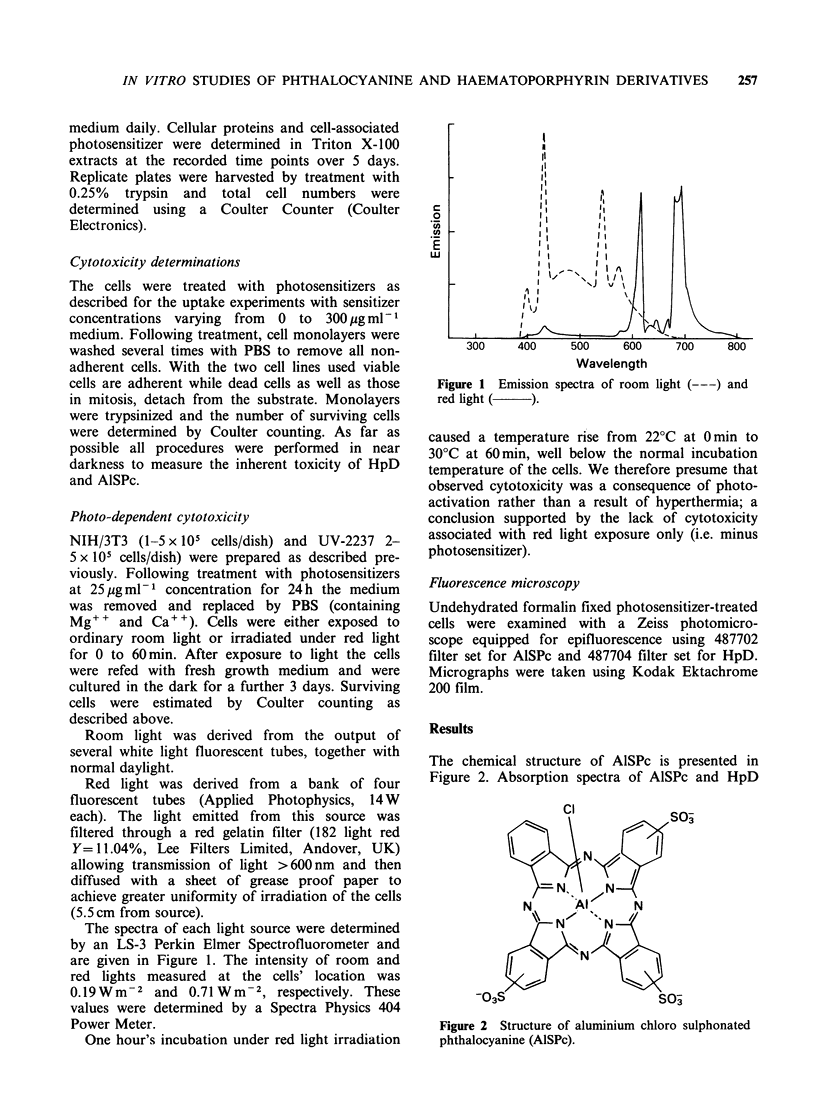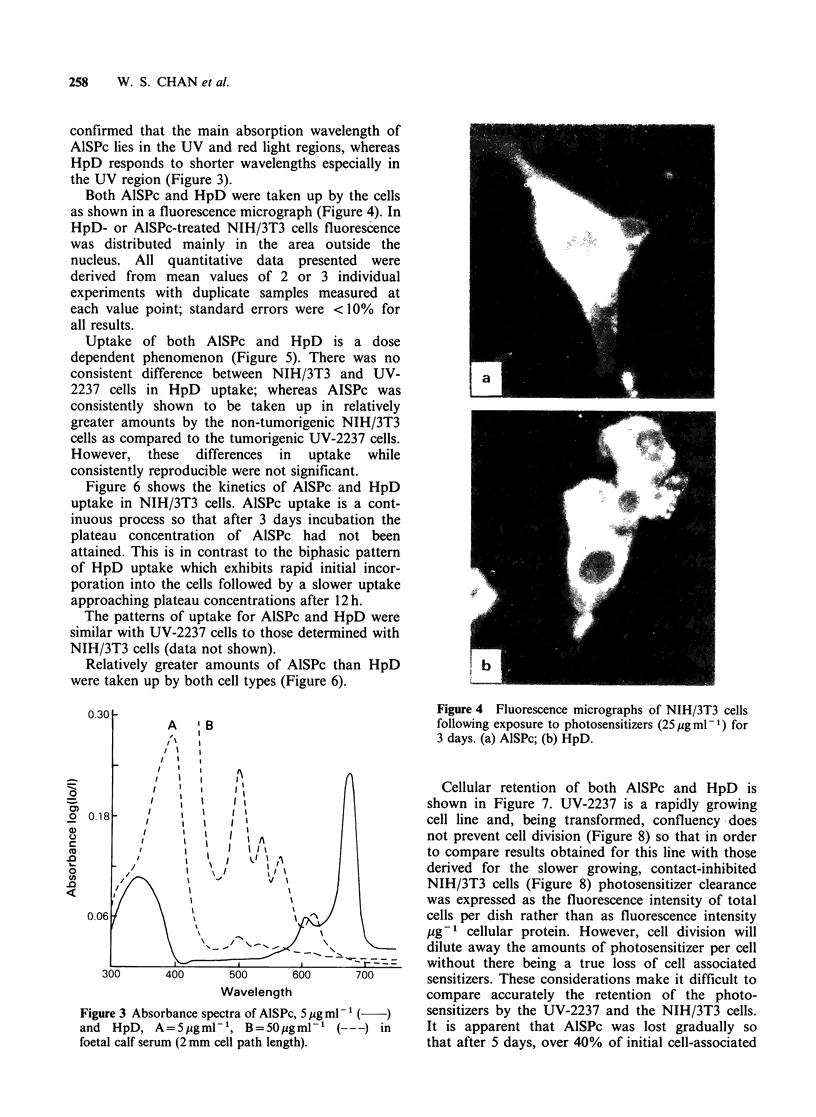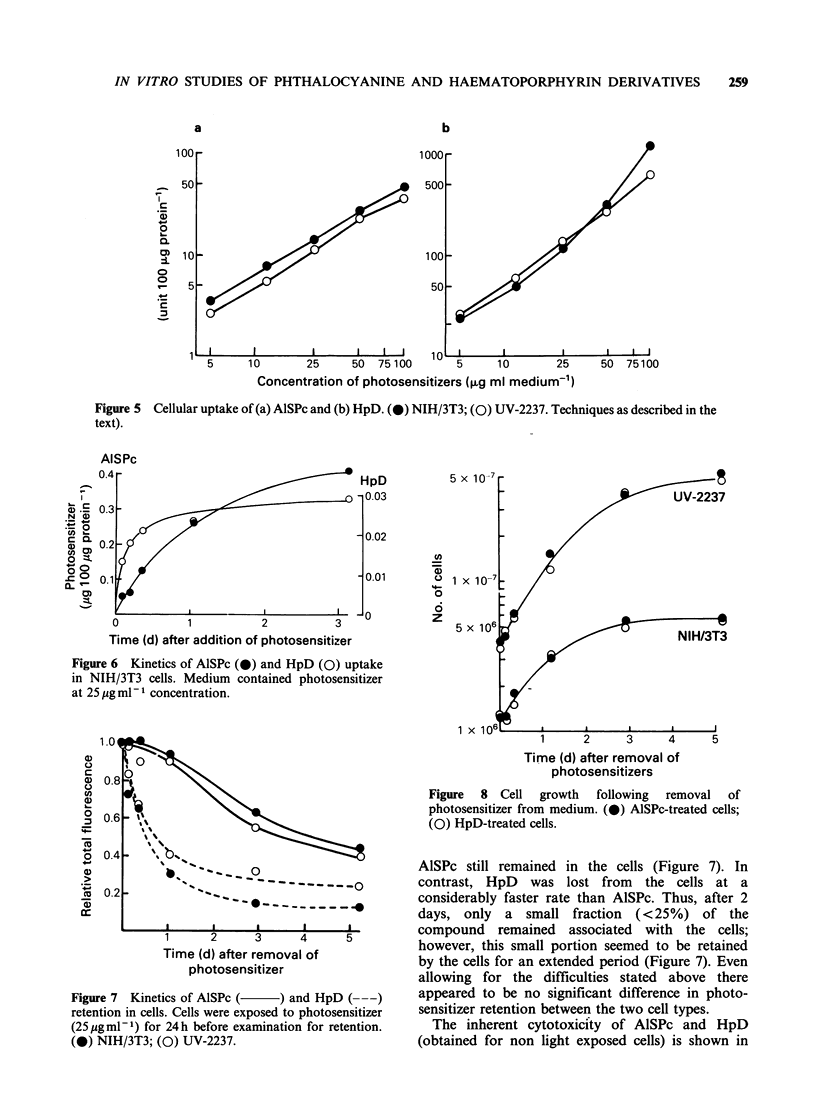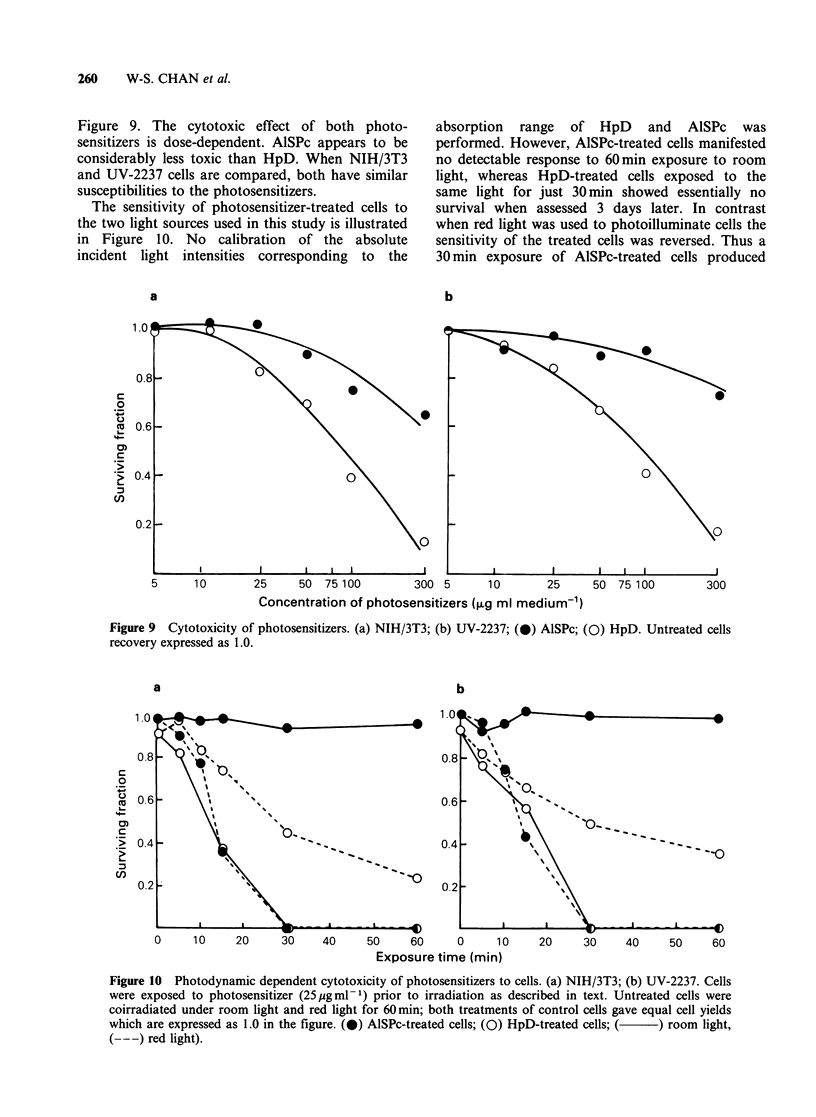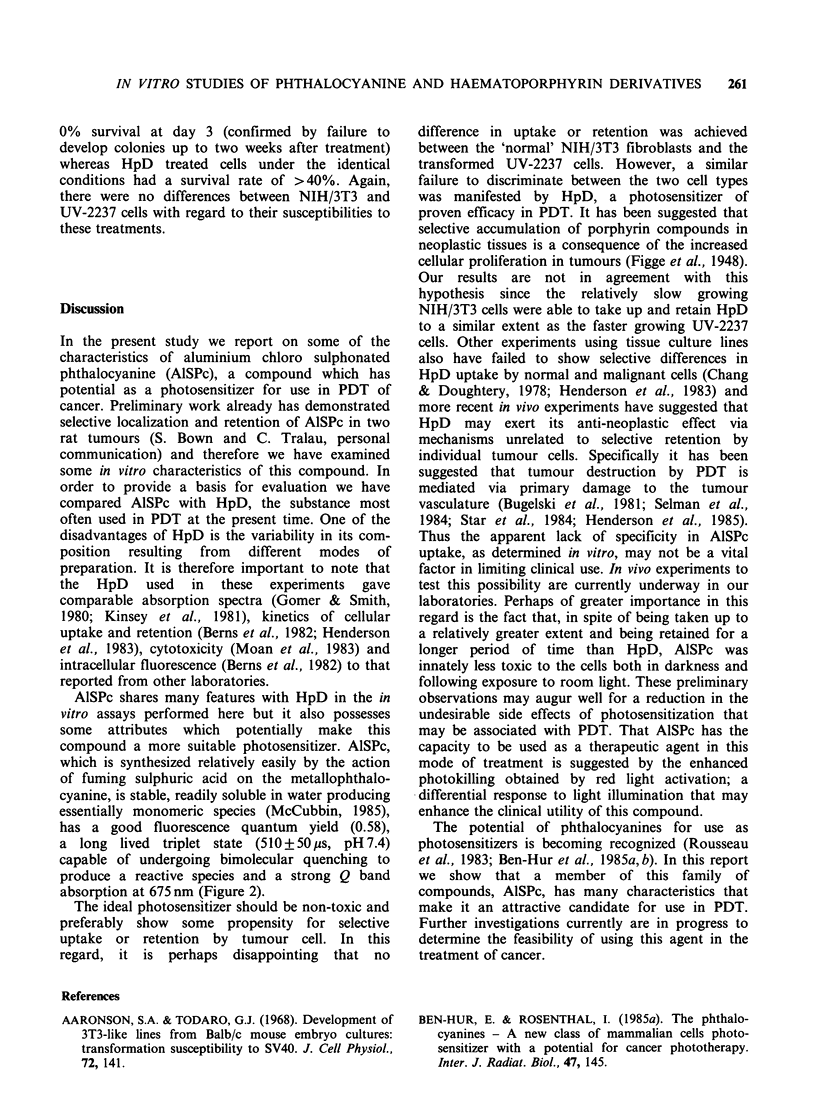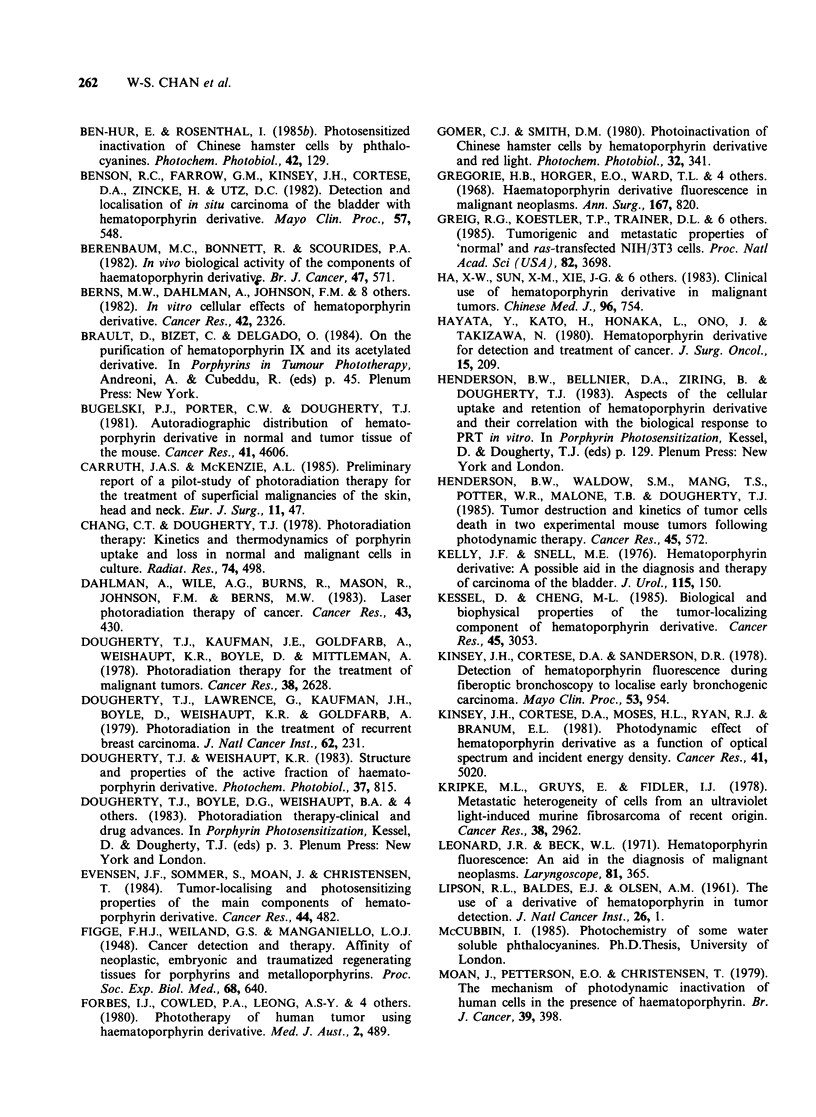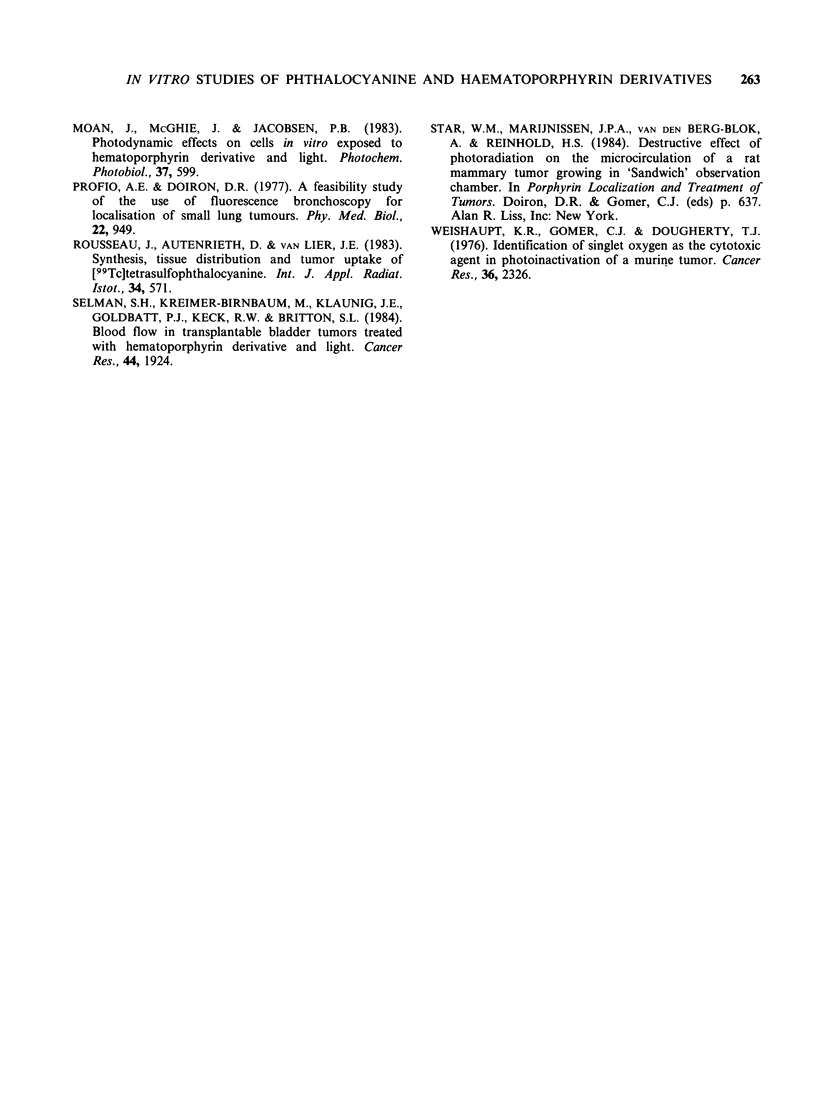# Cell uptake, distribution and response to aluminium chloro sulphonated phthalocyanine, a potential anti-tumour photosensitizer.

**DOI:** 10.1038/bjc.1986.43

**Published:** 1986-02

**Authors:** W. S. Chan, R. Svensen, D. Phillips, I. R. Hart

## Abstract

**Images:**


*Br. J. Cancer* (1986), **53**, 255–263

### Cell uptake, distribution and response to aluminium chloro sulphonated phthalocyanine, a potential anti-tumour photosensitizer

W-S. Chan[1], R. Svensen[2], D. Phillips[2] & I.R. Hart[1]

[1]*Imperial Cancer Research Fund Laboratories, P.O. Box 123, Lincoln's Inn Fields, London, WC2A 3PX and*
[2]*Royal Institution of Great Britain, 21 Albemarle Street, London, W1X 4BS, UK.*

**Summary** The uptake, retention and effects of aluminium chloro sulphonated phthalocyanine (AlSPc) were measured in two cell lines, UV-2237 a murine fibrosarcoma and the non-tumorigenic NIH/3T3 fibroblast line. The behaviour of cells treated with AlSPc was compared with that of those treated with haematoporphyrin derivative (HpD), a photosensitizer often used in photodynamic therapy (PDT) of cancer.

AlSPc absorbs light strongly in the red region, is taken up by cells in a dose dependent fashion and is retained *in vitro* over a period of days (5 days after exposure >40% remains cell-associated *versus* <25% of HpD). Additionally AlSPc was less cytotoxic to cells, maintained in darkness or exposed to room light, compared to HpD (100% viability versus 0% viability 3 days after 60 min exposure to room light). However red light (~600–700 nm) caused greater toxicity in AlSPc-treated cells (100%) than in similarly exposed HpD-treated cells (<60%). No significant differences were detected between the responses of the fibrosarcoma and the fibroblast cell lines. These characteristics of AlSPc suggest that it may prove to be a useful photosensitizer for PDT of cancer and this possibility is discussed.

Photodynamic therapy (PDT) of cancer is a relatively new technique that has been used clinically for the treatment of a variety of neoplasms. The basis of the technique is the systemic administration of photosensitizing dyes which localize to, or are retained preferentially by, the neoplastic lesion; the tumour is then exposed to light of the appropriate wavelength which photo-activates the dye leading to the generation of cyto-toxic products. Selectivity of effect is a consequence of both the preferential accumulation of the dye and the accuracy of light delivery.

At the present time the photosensitizer of choice in PDT is haematoporphyrin derivative (HpD) which shows preferential tumour localizing ability in a wide variety of human tumours (Lipson *et al.*, 1961; Gregorie *et al.*, 1968; Leonard & Beck, 1971; Profio & Doiron, 1977; Kinsey *et al.*, 1978; Benson *et al.*, 1982). The tumoricidal effect of HpD following light exposure is generally attributed to the formation of singlet oxygen generated by the transfer of energy from the excited HpD to tissue oxygen (Weishaupt *et al.*, 1976; Moan *et al.*, 1979). While the reasons for the preferential accumulation of HpD in neoplastic tissue, and even the exact basis of its anti-tumour effects, remain to be elucidated it has been used in the treatment of human cancer (Kelly & Snell, 1976; Dougherty *et al.*, 1978; Dougherty *et al.*, 1979; Forbes *et al.*, 1980; Hayata *et al.*, 1980; Dahlman *et al.*, 1983; Ha *et al.*, 1983; Carruth *et al.*, 1985). However, HpD has many characteristics which make it less than an ideal photosensitizer. HpD is formed by the treatment of haematoporphyrin with a mixture of acetic and sulphuric acid (Lipson *et al.*, 1961); a procedure which gives rise to a number of components. Several investigators have tried to determine the active component of HpD, and several candidate substances have been identified (Berenbaum *et al.*, 1982; Dougherty & Weishaupt, 1983; Evensen *et al.*, 1984; Kessel & Cheng, 1985). However, these components have not been chemically purified. The disadvantages of attempting to work clinically with a complex mixture of substances, whose composition may vary from batch to batch, are compounded by the fact that HpD may aggregate to different extents in different environments with resultant alterations in biological efficacy (Dougherty *et al.*, 1983). Furthermore, HpD absorbs red light poorly. This is a major disadvantage in treatment where it is desirable to use red light (absorbance 600–700 nm) for optimal tissue penetration. Finally, an additional complication is that patients receiving HpD for PDT often suffer from photosensitive reactions to daylight as a consequence of the retention of appreciable amounts of dye in the skin (Dougherty *et al.*, 1979).

These limitations have, in part, restricted the development of PDT and underlay the search for alternative photosensitive compounds for clinical

Correspondence: W-S. Chan
Received 2 September 1985; in final form, 15 October 1985.

© The Macmillan Press Ltd., 1986

use. In the present study we report on some of the characteristics of aluminium chloro sulphonated phthalocyanine (AlSPc), whose effects we have examined in tissue culture and compared to the more commonly used HpD.

### Materials and methods

### *Preparation of photosensitizers*

*Aluminium chloro sulphonated phthalocyanine (AlSPc)* AlSPc was obtained from Ciba-Geigy Dyestuffs and Chemicals (Postfach, Basel, Switzerland) and used as received. Sulphonation was reported to have been achieved by the action of fuming sulphuric acid on the metallophthalocyanine resulting in an average of three sulphonic acid groups per molecule (McCubbin, 1985). Isomers of this compound may be present and work is in progress to simplify this mixture though in molecular terms this substance is more homogeneous than HpD.

A stock solution was prepared by dissolving the compound in phosphate buffered saline (PBS) at 5 mg ml$^{-1}$ concentration. Aliquots were stored below 10°C in the dark, and added to growth medium to a final concentration immediately prior to use; this final solution was filtered, for sterility, through a 0.2 $\mu$m filter (Nalgene).

*Haematoporphyrin derivative (HpD)* Haematoporphyrin was obtained in its impure state from Sigma Chemical Company, purified by standard techniques (Brault *et al.*, 1984) and was treated with acetic and sulphuric acid as outlined by Lipson *et al.*, 1961. Once made the compound was dried and stored as a solid below 0°C in the dark.

Stock solutions were prepared by dissolving the final product in 0.1 N NaOH, pH was adjusted to 7.4 with 0.1 NHCl and PBS was added to 5 mg ml$^{-1}$ concentration. Solutions were stored below 10°C in the dark and brought to a final concentration and treated exactly as described for AlSPc.

### *Cell lines and culture conditions*

Two murine cell lines, NIH/3T3 and UV-2237, were used in this study. NIH/3T3 cells, derived from a Balb/c mouse, generally are considered to be non-tumorigenic (Aaronson & Todaro, 1968) though tumorigenic conversion may occur following prolonged cultivation in tissue culture (Greig *et al.*, 1985). UV-2237 is an ultraviolet light-induced fibrosarcoma of C3H/He mice (Kripke *et al.*, 1978). Both lines were grown on tissue culture plastic in E-4 growth medium, supplemented with 10% heat-inactivated foetal calf serum, at 37°C in 5% $CO_2$ and a humidified atmosphere. Because of differences in cell size the larger NIH/3T3 cells were grown in 90 mm petri dishes (Nunc) with 7–10 ml medium whereas UV-2237 were grown in 55 mm petri dishes containing 3–4 ml medium.

### *Photosensitizer uptake studies*

Cells (NIH/3T3, $1.5 \times 10^5 - 7.5 \times 10^5$; UV-2237, $3 \times 10^5 - 1.5 \times 10^6$) were plated in growth medium overnight for attachment. The next day medium was aspirated off and the cells were refed with medium containing either AlSPc or HpD at concentrations varying from 5 $\mu$g to 100 $\mu$g ml$^{-1}$. All procedures were performed in as close to complete darkness as possible with the laminar-flow hood enclosed within a dark curtain and light provided by a single bunsen-burner. When stored in incubators, petri dishes containing cells were protected from light by wrapping in aluminium foil.

After 3 days' growth adherent cells were washed 3 times with PBS. Proteins and cell-associated sensitizers were extracted with either 0.1 N NaOH (5–10 ml/dish) directly from the cell monolayer or with 0.5% Triton X-100 (in 10 mM Tris buffer, pH 8.3) from a trypsinized cell pellet. Triton X-100 extracts subsequently were subjected to sonication for two minutes. Both extraction procedures gave essentially identical results (data not shown). Both NaOH and Triton X-100 extracts were stored at −20°C before use. Protein was estimated by the Lowry method and fluorescence intensity was measured in a Perkin Elmer MPF-5 spectrofluorometer using these settings: AlSPc, excitation, 350–360 nm; emision, 670–680 nm. HpD, excitation, 395–405 nm; emission, 615–625 nm. Two dishes were used per time point in an experiment and individual values presented were obtained from the mean of 2–3 separate experiments. The amount of cell-associated photosensitizer was expressed as fluorescence units per 100 $\mu$g of cellular protein. Over the protein concentrations used in our studies we were unable to demonstrate quenching of any values obtained in a standard curve and thus assume that fluorescence is a measure of dye concentration.

The kinetics of cellular uptake of AlSPc and HpD were determined with NIH/3T3 cells. Cells plated as detailed above were then exposed to the photosensitizers at a concentration of 25 $\mu$g ml$^{-1}$ medium. At the recorded time points cells were extracted with NaOH and protein and fluorescence intensity were measured as described above.

### *Cellular retention of photosensitizers*

NIH/3T3 ($7.5 \times 10^5$) and UV-2237 ($1.5 \times 10^6$) cells were treated with photosensitizers at a concentration of 25 $\mu$g ml$^{-1}$ medium. After 24 h, cells were washed with PBS and refed with sensitizer-free medium daily. Cellular proteins and cell-associated photosensitizer were determined in Triton X-100 extracts at the recorded time points over 5 days. Replicate plates were harvested by treatment with 0.25% trypsin and total cell numbers were determined using a Coulter Counter (Coulter Electronics).

*Cytotoxicity determinations*

The cells were treated with photosensitizers as described for the uptake experiments with sensitizer concentrations varying from 0 to 300 $\mu g\,ml^{-1}$ medium. Following treatment, cell monolayers were washed several times with PBS to remove all non-adherent cells. With the two cell lines used viable cells are adherent while dead cells as well as those in mitosis, detach from the substrate. Monolayers were trypsinized and the number of surviving cells were determined by Coulter counting. As far as possible all procedures were performed in near darkness to measure the inherent toxicity of HpD and AlSPc.

*Photo-dependent cytotoxicity*

NIH/3T3 ($1-5 \times 10^5$ cells/dish) and UV-2237 $2-5 \times 10^5$ cells/dish) were prepared as described previously. Following treatment with photosensitizers at 25 $\mu g\,ml^{-1}$ concentration for 24 h the medium was removed and replaced by PBS (containing $Mg^{++}$ and $Ca^{++}$). Cells were either exposed to ordinary room light or irradiated under red light for 0 to 60 min. After exposure to light the cells were refed with fresh growth medium and were cultured in the dark for a further 3 days. Surviving cells were estimated by Coulter counting as described above.

Room light was derived from the output of several white light fluorescent tubes, together with normal daylight.

Red light was derived from a bank of four fluorescent tubes (Applied Photophysics, 14 W each). The light emitted from this source was filtered through a red gelatin filter (182 light red $Y = 11.04\%$, Lee Filters Limited, Andover, UK) allowing transmission of light $>600$ nm and then diffused with a sheet of grease proof paper to achieve greater uniformity of irradiation of the cells (5.5 cm from source).

The spectra of each light source were determined by an LS-3 Perkin Elmer Spectrofluorometer and are given in Figure 1. The intensity of room and red lights measured at the cells' location was 0.19 $W\,m^{-2}$ and 0.71 $W\,m^{-2}$, respectively. These values were determined by a Spectra Physics 404 Power Meter.

One hour's incubation under red light irradiation

**Figure 1** Emission spectra of room light (---) and red light (———).

caused a temperature rise from 22°C at 0 min to 30°C at 60 min, well below the normal incubation temperature of the cells. We therefore presume that observed cytotoxicity was a consequence of photoactivation rather than a result of hyperthermia; a conclusion supported by the lack of cytotoxicity associated with red light exposure only (i.e. minus photosensitizer).

*Fluorescence microscopy*

Undehydrated formalin fixed photosensitizer-treated cells were examined with a Zeiss photomicroscope equipped for epifluorescence using 487702 filter set for AlSPc and 487704 filter set for HpD. Micrographs were taken using Kodak Ektachrome 200 film.

### Results

The chemical structure of AlSPc is presented in Figure 2. Absorption spectra of AlSPc and HpD

**Figure 2** Structure of aluminium chloro sulphonated phthalocyanine (AlSPc).

confirmed that the main absorption wavelength of AlSPc lies in the UV and red light regions, whereas HpD responds to shorter wavelengths especially in the UV region (Figure 3).

Both AlSPc and HpD were taken up by the cells as shown in a fluorescence micrograph (Figure 4). In HpD- or AlSPc-treated NIH/3T3 cells fluorescence was distributed mainly in the area outside the nucleus. All quantitative data presented were derived from mean values of 2 or 3 individual experiments with duplicate samples measured at each value point; standard errors were <10% for all results.

Uptake of both AlSPc and HpD is a dose dependent phenomenon (Figure 5). There was no consistent difference between NIH/3T3 and UV-2237 cells in HpD uptake; whereas AlSPc was consistently shown to be taken up in relatively greater amounts by the non-tumorigenic NIH/3T3 cells as compared to the tumorigenic UV-2237 cells. However, these differences in uptake while consistently reproducible were not significant.

Figure 6 shows the kinetics of AlSPc and HpD uptake in NIH/3T3 cells. AlSPc uptake is a continuous process so that after 3 days incubation the plateau concentration of AlSPc had not been attained. This is in contrast to the biphasic pattern of HpD uptake which exhibits rapid initial incorporation into the cells followed by a slower uptake approaching plateau concentrations after 12 h.

The patterns of uptake for AlSPc and HpD were similar with UV-2237 cells to those determined with NIH/3T3 cells (data not shown).

Relatively greater amounts of AlSPc than HpD were taken up by both cell types (Figure 6).

Figure 3 Absorbance spectra of AlSPc, 5 $\mu$g ml$^{-1}$ (——) and HpD, A = 5 $\mu$g ml$^{-1}$, B = 50 $\mu$g ml$^{-1}$ (---) in foetal calf serum (2 mm cell path length).

Figure 4 Fluorescence micrographs of NIH/3T3 cells following exposure to photosensitizers (25 $\mu$g ml$^{-1}$) for 3 days. (a) AlSPc; (b) HpD.

Cellular retention of both AlSPc and HpD is shown in Figure 7. UV-2237 is a rapidly growing cell line and, being transformed, confluency does not prevent cell division (Figure 8) so that in order to compare results obtained for this line with those derived for the slower growing, contact-inhibited NIH/3T3 cells (Figure 8) photosensitizer clearance was expressed as the fluorescence intensity of total cells per dish rather than as fluorescence intensity $\mu$g$^{-1}$ cellular protein. However, cell division will dilute away the amounts of photosensitizer per cell without there being a true loss of cell associated sensitizers. These considerations make it difficult to compare accurately the retention of the photosensitizers by the UV-2237 and the NIH/3T3 cells. It is apparent that AlSPc was lost gradually so that after 5 days, over 40% of initial cell-associated

**Figure 5** Cellular uptake of (a) AlSPc and (b) HpD. (●) NIH/3T3; (○) UV-2237. Techniques as described in the text).

**Figure 6** Kinetics of AlSPc (●) and HpD (○) uptake in NIH/3T3 cells. Medium contained photosensitizer at 25 μg ml$^{-1}$ concentration.

**Figure 7** Kinetics of AlSPc (———) and HpD (– – –) retention in cells. Cells were exposed to photosensitizer (25 μg ml$^{-1}$) for 24 h before examination for retention. (●) NIH/3T3; (○) UV-2237.

**Figure 8** Cell growth following removal of photosensitizer from medium. (●) AlSPc-treated cells; (○) HpD-treated cells.

AlSPc still remained in the cells (Figure 7). In contrast, HpD was lost from the cells at a considerably faster rate than AlSPc. Thus, after 2 days, only a small fraction (<25%) of the compound remained associated with the cells; however, this small portion seemed to be retained by the cells for an extended period (Figure 7). Even allowing for the difficulties stated above there appeared to be no significant difference in photosensitizer retention between the two cell types.

The inherent cytotoxicity of AlSPc and HpD (obtained for non light exposed cells) is shown in

Figure 9. The cytotoxic effect of both photosensitizers is dose-dependent. AlSPc appears to be considerably less toxic than HpD. When NIH/3T3 and UV-2237 cells are compared, both have similar susceptibilities to the photosensitizers.

The sensitivity of photosensitizer-treated cells to the two light sources used in this study is illustrated in Figure 10. No calibration of the absolute incident light intensities corresponding to the absorption range of HpD and AlSPc was performed. However, AlSPc-treated cells manifested no detectable response to 60 min exposure to room light, whereas HpD-treated cells exposed to the same light for just 30 min showed essentially no survival when assessed 3 days later. In contrast when red light was used to photoilluminate cells the sensitivity of the treated cells was reversed. Thus a 30 min exposure of AlSPc-treated cells produced

**Figure 9** Cytotoxicity of photosensitizers. (a) NIH/3T3; (b) UV-2237; (●) AlSPc; (○) HpD. Untreated cells recovery expressed as 1.0.

**Figure 10** Photodynamic dependent cytotoxicity of photosensitizers to cells. (a) NIH/3T3; (b) UV-2237. Cells were exposed to photosensitizer ($25 \mu g ml^{-1}$) prior to irradiation as described in text. Untreated cells were coirradiated under room light and red light for 60 min; both treatments of control cells gave equal cell yields which are expressed as 1.0 in the figure. (●) AlSPc-treated cells; (○) HpD-treated cells; (———) room light, (- - -) red light).

0% survival at day 3 (confirmed by failure to develop colonies up to two weeks after treatment) whereas HpD treated cells under the identical conditions had a survival rate of >40%. Again, there were no differences between NIH/3T3 and UV-2237 cells with regard to their susceptibilities to these treatments.

### Discussion

In the present study we report on some of the characteristics of aluminium chloro sulphonated phthalocyanine (AlSPc), a compound which has potential as a photosensitizer for use in PDT of cancer. Preliminary work already has demonstrated selective localization and retention of AlSPc in two rat tumours (S. Bown and C. Tralau, personal communication) and therefore we have examined some *in vitro* characteristics of this compound. In order to provide a basis for evaluation we have compared AlSPc with HpD, the substance most often used in PDT at the present time. One of the disadvantages of HpD is the variability in its composition resulting from different modes of preparation. It is therefore important to note that the HpD used in these experiments gave comparable absorption spectra (Gomer & Smith, 1980; Kinsey *et al.*, 1981), kinetics of cellular uptake and retention (Berns *et al.*, 1982; Henderson *et al.*, 1983), cytotoxicity (Moan *et al.*, 1983) and intracellular fluorescence (Berns *et al.*, 1982) to that reported from other laboratories.

AlSPc shares many features with HpD in the *in vitro* assays performed here but it also possesses some attributes which potentially make this compound a more suitable photosensitizer. AlSPc, which is synthesized relatively easily by the action of fuming sulphuric acid on the metallophthalocyanine, is stable, readily soluble in water producing essentially monomeric species (McCubbin, 1985), has a good fluorescence quantum yield (0.58), a long lived triplet state ($510 \pm 50\,\mu s$, pH 7.4) capable of undergoing bimolecular quenching to produce a reactive species and a strong *Q* band absorption at 675 nm (Figure 2).

The ideal photosensitizer should be non-toxic and preferably show some propensity for selective uptake or retention by tumour cell. In this regard, it is perhaps disappointing that no difference in uptake or retention was achieved between the 'normal' NIH/3T3 fibroblasts and the transformed UV-2237 cells. However, a similar failure to discriminate between the two cell types was manifested by HpD, a photosensitizer of proven efficacy in PDT. It has been suggested that selective accumulation of porphyrin compounds in neoplastic tissues is a consequence of the increased cellular proliferation in tumours (Figge *et al.*, 1948). Our results are not in agreement with this hypothesis since the relatively slow growing NIH/3T3 cells were able to take up and retain HpD to a similar extent as the faster growing UV-2237 cells. Other experiments using tissue culture lines also have failed to show selective differences in HpD uptake by normal and malignant cells (Chang & Doughtery, 1978; Henderson *et al.*, 1983) and more recent *in vivo* experiments have suggested that HpD may exert its anti-neoplastic effect via mechanisms unrelated to selective retention by individual tumour cells. Specifically it has been suggested that tumour destruction by PDT is mediated via primary damage to the tumour vasculature (Bugelski *et al.*, 1981; Selman *et al.*, 1984; Star *et al.*, 1984; Henderson *et al.*, 1985). Thus the apparent lack of specificity in AlSPc uptake, as determined *in vitro*, may not be a vital factor in limiting clinical use. *In vivo* experiments to test this possibility are currently underway in our laboratories. Perhaps of greater importance in this regard is the fact that, in spite of being taken up to a relatively greater extent and being retained for a longer period of time than HpD, AlSPc was innately less toxic to the cells both in darkness and following exposure to room light. These preliminary observations may augur well for a reduction in the undesirable side effects of photosensitization that may be associated with PDT. That AlSPc has the capacity to be used as a therapeutic agent in this mode of treatment is suggested by the enhanced photokilling obtained by red light activation; a differential response to light illumination that may enhance the clinical utility of this compound.

The potential of phthalocyanines for use as photosensitizers is becoming recognized (Rousseau *et al.*, 1983; Ben-Hur *et al.*, 1985*a*, *b*). In this report we show that a member of this family of compounds, AlSPc, has many characteristics that make it an attractive candidate for use in PDT. Further investigations currently are in progress to determine the feasibility of using this agent in the treatment of cancer.

### References

AARONSON, S.A. & TODARO, G.J. (1968). Development of 3T3-like lines from Balb/c mouse embryo cultures: transformation susceptibility to SV40. *J. Cell Physiol.*, **72**, 141.

BEN-HUR, E. & ROSENTHAL, I. (1985*a*). The phthalocyanines – A new class of mammalian cells photosensitizer with a potential for cancer phototherapy. *Inter. J. Radiat. Biol.*, **47**, 145.

BEN-HUR, E. & ROSENTHAL, I. (1985*b*). Photosensitized inactivation of Chinese hamster cells by phthalocyanines. *Photochem. Photobiol.*, **42**, 129.

BENSON, R.C., FARROW, G.M., KINSEY, J.H., CORTESE, D.A., ZINCKE, H. & UTZ, D.C. (1982). Detection and localisation of *in situ* carcinoma of the bladder with hematoporphyrin derivative. *Mayo Clin. Proc.*, **57**, 548.

BERENBAUM, M.C., BONNETT, R. & SCOURIDES, P.A. (1982). *In vivo* biological activity of the components of haematoporphyrin derivative. *Br. J. Cancer*, **47**, 571.

BERNS, M.W., DAHLMAN, A., JOHNSON, F.M. & 8 others. (1982). *In vitro* cellular effects of hematoporphyrin derivative. *Cancer Res.*, **42**, 2326.

BRAULT, D., BIZET, C. & DELGADO, O. (1984). On the purification of hematoporphyrin IX and its acetylated derivative. In *Porphyrins in Tumour Phototherapy*, Andreoni, A. & Cubeddu, R. (eds) p. 45. Plenum Press: New York.

BUGELSKI, P.J., PORTER, C.W. & DOUGHERTY, T.J. (1981). Autoradiographic distribution of hematoporphyrin derivative in normal and tumor tissue of the mouse. *Cancer Res.*, **41**, 4606.

CARRUTH, J.A.S. & McKENZIE, A.L. (1985). Preliminary report of a pilot-study of photoradiation therapy for the treatment of superficial malignancies of the skin, head and neck. *Eur. J. Surg.*, **11**, 47.

CHANG, C.T. & DOUGHERTY, T.J. (1978). Photoradiation therapy: Kinetics and thermodynamics of porphyrin uptake and loss in normal and malignant cells in culture. *Radiat. Res.*, **74**, 498.

DAHLMAN, A., WILE, A.G., BURNS, R., MASON, R., JOHNSON, F.M. & BERNS, M.W. (1983). Laser photoradiation therapy of cancer. *Cancer Res.*, **43**, 430.

DOUGHERTY, T.J., KAUFMAN, J.E., GOLDFARB, A., WEISHAUPT, K.R., BOYLE, D. & MITTLEMAN, A. (1978). Photoradiation therapy for the treatment of malignant tumors. *Cancer Res.*, **38**, 2628.

DOUGHERTY, T.J., LAWRENCE, G., KAUFMAN, J.H., BOYLE, D., WEISHAUPT, K.R. & GOLDFARB, A. (1979). Photoradiation in the treatment of recurrent breast carcinoma. *J. Natl Cancer Inst.*, **62**, 231.

DOUGHERTY, T.J. & WEISHAUPT, K.R. (1983). Structure and properties of the active fraction of haematoporphyrin derivative. *Photochem. Photobiol.*, **37**, 815.

DOUGHERTY, T.J., BOYLE, D.G., WEISHAUPT, B.A. & 4 others. (1983). Photoradiation therapy-clinical and drug advances. In *Porphyrin Photosensitization*, Kessel, D. & Dougherty, T.J. (eds) p. 3. Plenum Press: New York and London.

EVENSEN, J.F., SOMMER, S., MOAN, J. & CHRISTENSEN, T. (1984). Tumor-localising and photosensitizing properties of the main components of hematoporphyrin derivative. *Cancer Res.*, **44**, 482.

FIGGE, F.H.J., WEILAND, G.S. & MANGANIELLO, L.O.J. (1948). Cancer detection and therapy. Affinity of neoplastic, embryonic and traumatized regenerating tissues for porphyrins and metalloporphyrins. *Proc. Soc. Exp. Biol. Med.*, **68**, 640.

FORBES, I.J., COWLED, P.A., LEONG, A.S-Y. & 4 others. (1980). Phototherapy of human tumor using haematoporphyrin derivative. *Med. J. Aust.*, **2**, 489.

GOMER, C.J. & SMITH, D.M. (1980). Photoinactivation of Chinese hamster cells by hematoporphyrin derivative and red light. *Photochem. Photobiol.*, **32**, 341.

GREGORIE, H.B., HORGER, E.O., WARD, T.L. & 4 others. (1968). Haematoporphyrin derivative fluorescence in malignant neoplasms. *Ann. Surg.*, **167**, 820.

GREIG, R.G., KOESTLER, T.P., TRAINER, D.L. & 6 others. (1985). Tumorigenic and metastatic properties of 'normal' and *ras*-transfected NIH/3T3 cells. *Proc. Natl Acad. Sci (USA)*, **82**, 3698.

HA, X-W., SUN, X-M., XIE, J-G. & 6 others. (1983). Clinical use of hematoporphyrin derivative in malignant tumors. *Chinese Med. J.*, **96**, 754.

HAYATA, Y., KATO, H., HONAKA, L., ONO, J. & TAKIZAWA, N. (1980). Hematoporphyrin derivative for detection and treatment of cancer. *J. Surg. Oncol.*, **15**, 209.

HENDERSON, B.W., BELLNIER, D.A., ZIRING, B. & DOUGHERTY, T.J. (1983). Aspects of the cellular uptake and retention of hematoporphyrin derivative and their correlation with the biological response to PRT *in vitro*. In *Porphyrin Photosensitization*, Kessel, D. & Dougherty, T.J. (eds) p. 129. Plenum Press: New York and London.

HENDERSON, B.W., WALDOW, S.M., MANG, T.S., POTTER, W.R., MALONE, T.B. & DOUGHERTY, T.J. (1985). Tumor destruction and kinetics of tumor cells death in two experimental mouse tumors following photodynamic therapy. *Cancer Res.*, **45**, 572.

KELLY, J.F. & SNELL, M.E. (1976). Hematoporphyrin derivative: A possible aid in the diagnosis and therapy of carcinoma of the bladder. *J. Urol.*, **115**, 150.

KESSEL, D. & CHENG, M-L. (1985). Biological and biophysical properties of the tumor-localizing component of hematoporphyrin derivative. *Cancer Res.*, **45**, 3053.

KINSEY, J.H., CORTESE, D.A. & SANDERSON, D.R. (1978). Detection of hematoporphyrin fluorescence during fiberoptic bronchoscopy to localise early bronchogenic carcinoma. *Mayo Clin. Proc.*, **53**, 954.

KINSEY, J.H., CORTESE, D.A., MOSES, H.L., RYAN, R.J. & BRANUM, E.L. (1981). Photodynamic effect of hematoporphyrin derivative as a function of optical spectrum and incident energy density. *Cancer Res.*, **41**, 5020.

KRIPKE, M.L., GRUYS, E. & FIDLER, I.J. (1978). Metastatic heterogeneity of cells from an ultraviolet light-induced murine fibrosarcoma of recent origin. *Cancer Res.*, **38**, 2962.

LEONARD, J.R. & BECK, W.L. (1971). Hematoporphyrin fluorescence: An aid in the diagnosis of malignant neoplasms. *Laryngoscope*, **81**, 365.

LIPSON, R.L., BALDES, E.J. & OLSEN, A.M. (1961). The use of a derivative of hematoporphyrin in tumor detection. *J. Natl Cancer Inst.*, **26**, 1.

McCUBBIN, I. (1985). Photochemistry of some water soluble phthalocyanines. Ph.D.Thesis, University of London.

MOAN, J., PETTERSON, E.O. & CHRISTENSEN, T. (1979). The mechanism of photodynamic inactivation of human cells in the presence of haematoporphyrin. *Br. J. Cancer*, **39**, 398.

MOAN, J., McGHIE, J. & JACOBSEN, P.B. (1983). Photodynamic effects on cells *in vitro* exposed to hematoporphyrin derivative and light. *Photochem. Photobiol.*, **37**, 599.

PROFIO, A.E. & DOIRON, D.R. (1977). A feasibility study of the use of fluorescence bronchoscopy for localisation of small lung tumours. *Phy. Med. Biol.*, **22**, 949.

ROUSSEAU, J., AUTENRIETH, D. & VAN LIER, J.E. (1983). Synthesis, tissue distribution and tumor uptake of [$^{99}$Tc]tetrasulfophthalocyanine. *Int. J. Appl. Radiat. Istot.*, **34**, 571.

SELMAN, S.H., KREIMER-BIRNBAUM, M., KLAUNIG, J.E., GOLDBATT, P.J., KECK, R.W. & BRITTON, S.L. (1984). Blood flow in transplantable bladder tumors treated with hematoporphyrin derivative and light. *Cancer Res.*, **44**, 1924.

STAR, W.M., MARIJNISSEN, J.P.A., VAN DEN BERG-BLOK, A. & REINHOLD, H.S. (1984). Destructive effect of photoradiation on the microcirculation of a rat mammary tumor growing in 'Sandwich' observation chamber. In *Porphyrin Localization and Treatment of Tumors*. Doiron, D.R. & Gomer, C.J. (eds) p. 637. Alan R. Liss, Inc: New York.

WEISHAUPT, K.R., GOMER, C.J. & DOUGHERTY, T.J. (1976). Identification of singlet oxygen as the cytotoxic agent in photoinactivation of a murine tumor. *Cancer Res.*, **36**, 2326.